# Distal femoral shortening osteotomy for treatment of sciatic nerve palsy after total hip arthroplasty — a report of 3 cases

**DOI:** 10.1080/17453674.2018.1520679

**Published:** 2018-10-01

**Authors:** Benjamin Puliero, William G Blakeney, Yann Beaulieu, Alain Roy, Pascal-André Vendittoli

**Affiliations:** 1 Department of Surgery, CIUSSS-de-L’Est-de-L’Ile-de-Montréal, Hôpital Maisonneuve Rosemont. Montréal, Québec;;; 2 Department of Surgery, Université de Montréal, Québec, Canada

Total hip arthroplasty (THA) for developmental dysplasia of the hip (DDH) with associated femoral head dislocation is a challenging procedure, with a high complication rate. These include dislocation, nonunion or malunion of osteotomies, infection, implant loosening, and sciatic nerve injury (Rogers et al. 2012, Sonohata et al. 2016). In severe DDH cases, overlengthening of the lower limb may cause sciatic nerve injury, a devastating complication (De Fine et al. 2017). Secondary neurologic pain and associated muscle weakness may overshadow otherwise excellent arthroplasty reconstruction and lead to patient dissatisfaction. In order to avoid this complication, it has been suggested that a proximal or subtrochanteric femoral osteotomy be performed when lengthening exceeds 4 cm (Cameron et al. 1998). Other authors have suggested intraoperative monitoring to assess nerve function as a decision tool to determine the maximal or appropriate limb lengthening (Paavilainen 1997). When a sciatic nerve injury is identified postoperatively, there is debate over what is the optimal treatment.

In 3 THA cases for DDH (2 patients), we describe an effective surgical technique for treating associated sciatic nerve injuries secondary to excessive nerve tension. A late shortening distal femoral osteotomy was performed as treatment for 2 hips and as a prophylactic procedure for the third case.

## Surgical technique

The patient is placed in the supine position. The distal femur is exposed using a lateral approach. A Kirschner wire is inserted under fluoroscopic guidance and is used as a guide to create a path for the saw blade. The bone is scored longitudinally to mark femoral rotation. The osteotomy is then performed and the desired length of bone removed. The 2 bone segments are approximated and a 4-hole 90° blade-plate applied with a compression guide. Postoperatively, the patient is allowed partial weight-bearing initially, increasing to full weight-bearing at 12 weeks.

## Patient 1

**Figure 1.**
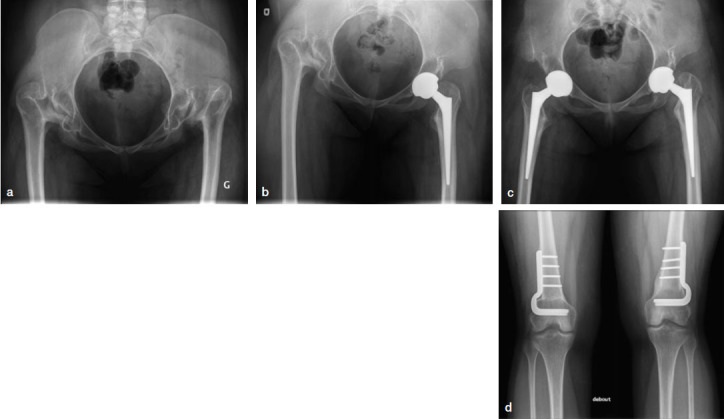
Case 1 with bilateral developmental dysplasia of the hip. a. Preoperativaely b. Postoperatively, THA with 5.5 cm leg lengthening. c. Bilateral THAs. d. Healed bilateral distal femoral 3 cm shortening osteotomies.

An 18-year-old woman with bilateral DDH was referred to our arthroplasty clinic because of bilateral groin pain and increasing difficulty with walking. Radiographs showed bilateral DDH type III Crowe classification (Figure 1a). There was no leg length discrepancy due to bilateral pathology. On preoperative templating, by placing the acetabular component in the true acetabulum, planned limb lengthening was 4 cm.

The THA was performed using a posterior approach. A 42 mm monobloc acetabular cup (Zimmer Maxera; Zimmer Biomet, Warsaw, IN, USA) was placed in the true acetabulum to restore the hip center of rotation. The cup used is a monobloc, uncemented implant with a non-modular ceramic liner (Biolox Delta; CeramTec, Plochingen, Germany). Matching ceramic femoral head size was 32 mm. With medialization, cup coverage was sufficient without the requirement for bone graft. The femoral stem was a straight conical uncemented stem (Zimmer Wagner cone prosthesis).

During the procedure, the surgeon (AR) noted a tight sciatic nerve, but thought it was acceptable and no femoral shortening was performed. Neither electromyographic monitoring nor wake-up tests are commonly used in our practice. On the day following surgery, the patient had paresthesia in the sciatic nerve distribution but motor function was intact. The postoperative radiographs (Figure 1b) showed a 5.5 cm leg lengthening which was thought to be the cause of the sciatic nerve paresthesia. As there was no motor palsy, the initial diagnosis was a neurapraxia. Therefore, the patient was monitored to see if there was any recovery of nerve function. During the next 5 weeks, there was no improvement of the symptoms with a persistence of paresthesia when the knee was in full extension. A diagnosis of axonotmesis was made and the decision was taken to perform a femoral osteotomy to reduce the nerve tension and potentially favor nerve recovery. To avoid compromising the stability of the hip reconstruction surgery, a distal femoral osteotomy was seen as a better option.

At 6 weeks after the initial surgery, the distal femoral osteotomy procedure was performed (3 cm shortening). Within the immediate days after the osteotomy, there was a significant reduction in pain and paresthesia. Complete recovery of sciatic nerve function was obtained within 6 weeks. The osteotomy was fully united by 3 -months. At 6-year follow-up, the patient remains asymptomatic with excellent hip function, no pain or dislocation, and normal motion.

2 years after the left THA, the patient requested surgery for her right hip. At the time of surgery on that side, a distal femoral osteotomy was performed in the same setting, immediately prior to the THA (3 cm shortening). The THA was uncomplicated, with importantly no sciatic nerve problems postoperatively (Figures 1c and d).

## Patient 2

A 35-year-old woman suffering from unilateral left DDH was referred to the same surgeon (AR). A THA was performed without using a shortening osteotomy.

In the recovery room during the postoperative examination, a sciatic nerve palsy was diagnosed with a complete motor palsy. An excessive 6 cm lower limb lengthening was diagnosed to be the cause of the injury. The surgeon decided to take the patient back to the operating room the following day. The previously described distal femoral osteotomy technique was performed (shortening of 3 cm). 2 days after the osteotomy, there was complete recovery of the motor and sensory function of the sciatic nerve. At 2 years’ follow-up, there was complete union of the osteotomy and no functional consequences of the transient nerve injury.

## Discussion

Sciatic nerve palsy is a relatively common complication after THA in patients with DDH. When it occurs, the best treatment remains controversial. To our knowledge, this article is the first description of a distal femoral osteotomy performed for the treatment of a sciatic nerve injury after THA. This strategy resulted in successful treatment of sciatic nerve injuries in 2 THAs and was as an effective prophylactic option in another.

One of the causes for sciatic nerve injury is excessive leg lengthening during THA. Higuchi et al. (2015) reported that exceeding 5 cm of lengthening during THA is a risk factor for sciatic nerve injury. In their case series of high dysplasia of the hip, Zappe et al. (2014) reported that only 17 of 34 patients fully recovered from their nerve lesions. They noted that recovery of function was still possible after 2 years, but only in 3 out of 17 of their patients.

Neurapraxia lesions are known to recover within 3–4 weeks. When there is no recovery after 1 month, axonotmesis or neurotmesis is present (Campbell 2008). When an acute causative element such as overlengthening is present, we do not recommend waiting for a formal diagnosis of axonotmesis. Intervention should be performed as soon as possible. Interestingly, our first case demonstrates that full recovery is still possible even with a late intervention (6 weeks).

Our observations have some limitations. The few cases described do not permit us to recommend this technique for every case of DDH requiring leg lengthening. In our practice, leg lengthening when performing a THA should be a maximum of 3–4 cm, but sometimes errors occur during surgery that may result in the requirement for the described distal femoral shortening osteotomy. This solution should be kept as a preventive or salvage procedure, since we do not have sufficient data to support more widespread use presently.

One of the surgical options for sciatic nerve palsy after THA is surgical neurolysis. Regev et al. (2015) showed that when no improvement was found after 6 months of observation, surgical neurolysis improved function and pain outcomes. Another study by Chughtai et al. (2017) compared patients who underwent nerve decompression treatment with those who did not. Their results showed a significant improvement in motor strength and pain when a surgical decompression was performed. Their literature review demonstrated the benefit of surgical treatment (improvement in 75%) compared with non-operative treatment (improvement in 33%). In our cases the etiology was most certainly an excessive leg lengthening, and neurolysis without addressing the lengthening was deemed inappropriate.

In THA in high dysplasia of the hip, several techniques for proximal femoral osteotomies are described to reduce leg lengthening and tension on the sciatic nerve (Reikeras et al. 2010). Numerous complications have been reported with proximal or subtrochanteric osteotomies such as nonunion, malunion, fractures (Paavilainen et al. 1993), and sciatic nerve palsy (Sener et al. 2002, Rollo et al. 2017). The advantage of performing a distal femoral osteotomy is that the stability of the hip is not compromised. Distal femoral shortening osteotomy concomitant with THA has been described by Koulouvaris et al. (2008) and provided satisfactory results regarding postoperative complications, with only 1 case of malunion and no cases of nonunion reported in 24 hips. Clinical outcomes were also excellent and comparable to proximal osteotomies. In their series, however, the mean shortening of the femur was only 18 mm (10–25). Their reported technique also differs from 2 of our cases, in that they performed their osteotomy at the time of THA, not as treatment for a sciatic nerve injury postoperatively.

Distal femoral shortening osteotomy also allows for correction of frontal knee deformity, which is often associated with DDH, by changing the cylinder shape of the removed piece of bone into a wedge. Cameron et al. (2015) reported that distal femoral opening-wedge osteotomy also improved knee pain and functional scores. Only 1 nonunion occurred in their series of 31 knees and no nerve injuries were reported. Distal femoral osteotomy as described in our surgical technique also avoids rotational malunion when performed.

Our technique has several advantages compared with proximal femoral osteotomy. First, the surgery is simple and there is no need to modify the hip arthroplasty, which could compromise stability. Second, it avoids the risk of infection or periprosthetic fracture due to revision surgery. Similar techniques have been used in the absence of sciatic nerve palsy with few complications reported (Cameron et al. 2015). It also avoids the risk of metaphyseal nonunion seen with proximal femoral osteotomies. Lastly, it may be an opportunity to correct severe genu valgum, which is often present in the DDH patient, by performing a wedge osteotomy.

When a sciatic nerve palsy is diagnosed after THA with an important leg lengthening, the use of distal femoral shortening osteotomy should be considered.

BP was involved in data collection and drafting of the manuscript. WB and YB were reviewers of the draft manuscript. AR was the senior surgeon performing the surgeries. PAV was the main reviewer and helped with critical appraisal of the manuscript.

Acta thanks Johan Kärrholm for help with peer review of this study.
